# Juveniles Are More Resistant to Warming than Adults in 4 Species of Antarctic Marine Invertebrates

**DOI:** 10.1371/journal.pone.0066033

**Published:** 2013-06-26

**Authors:** Lloyd S. Peck, Terri Souster, Melody S. Clark

**Affiliations:** British Antarctic Survey, High Cross, Cambridge, Cambridgeshire, United Kingdom; The Australian National University, Australia

## Abstract

Juvenile stages are often thought to be less resistant to thermal challenges than adults, yet few studies make direct comparisons using the same methods between different life history stages. We tested the resilience of juvenile stages compared to adults in 4 species of Antarctic marine invertebrate over 3 different rates of experimental warming. The species used represent 3 phyla and 4 classes, and were the soft-shelled clam *Laternula elliptica*, the sea cucumber *Cucumaria georgiana,* the sea urchin *Sterechinus neumayeri*, and the seastar *Odontaster validus*. All four species are widely distributed, locally abundant to very abundant and are amongst the most important in the ecosystem for their roles. At the slowest rate of warming used (1°C 3 days^−1^) juveniles survived to higher temperatures than adults in all species studied. At the intermediate rate (1°C day^−1^) juveniles performed better in 3 of the 4 species, with no difference in the 4^th^, and at the fastest rate of warming (1°C h^−1^) *L. elliptica* adults survived to higher temperatures than juveniles, but in *C. georgiana* juveniles survived to higher temperatures than adults and there were no differences in the other species. Oxygen limitation may explain the better performance of juveniles at the slower rates of warming, whereas the loss of difference between juveniles and adults at the fastest rate of warming suggests another mechanism sets the temperature limit here.

## Introduction

Early life stages of marine invertebrates have been considered to be vulnerable or fragile in relation to environmental stresses for over 100 years [1]-[3], with many species exhibiting high mortality during development and early growth and lower mortality rates with maturity [4], [5]. Many marine species produce large numbers of gametes, the vast majority of which fail to reach maturity. Furthermore warmer seas are expected to impact directly on development rates, which generally increase with temperature to an optimum, beyond which mortality levels increase rapidly [6]-[8]. However, there is relatively little data to either support or disprove the expectation of reduced physiological tolerance to temperature stress of juvenile stages.

Larval lobsters have been shown to be less tolerant to elevated temperature than adults [9]. Juveniles of the Dungeness crab, *Cancer magister* are less tolerant than larvae to elevated temperature in experiments, and this correlates with distribution limits [10]. Early juveniles of the snail *Nucella emarginata* are less capable of surviving elevated temperatures than later stages [11]. One proposed explanation for these findings is that the oxygen delivery system may not be fully developed in early life history stages and the oxygen limitation hypothesis, widely used to explain variations in thermal tolerance in aquatic species, and hence distributions (e.g. [12]), may produce tighter restrictions on early stages. Effects on distribution, however, may be complex, because several marine groups grow to larger size at low temperatures [13]. This has been explained as a consequence of oxygen availability [14], [15], or as a consequence of increased growth efficiency at lower temperature [16]. Increased temperature also raises growth, development and metabolic rates of juvenile stages up to a critical threshold. This increases energy and resource use which may cause a energy stress and failure due to food limitation, which has been used to explain poor recruitment in coral reef fish during warm El Nino events [17], [18].

Upper thermal limits (UTL) were shown to vary markedly in experiments with the rate of warming used across 14 species of Antarctic benthic marine invertebrates by [19]. They demonstrated that at faster rates of warming, UTLs were higher than at slower rates of warming. UTLs ranged from 8.2°C to 17.6°C at a warming rate of 1°C day^−1^, whereas these values were 1.5°C to 5.5°C when they were warmed at 1°C month^−1^. This work also analysed thermal limits in relation to activity and showed that more active species had higher UTL values than slow moving and sedentary species. There was no correlation with trophic guild, as sedentary predators such as anemones had low UTLs whereas active herbivorous amphipods had high values. These data supported the oxygen limitation paradigm as more active species have higher aerobic scopes than sedentary taxa [20].

Similar relationships of reduced temperature limit at slower rates of warming have been identified for temperate [21] and tropical [22] marine species. [21] further demonstrated that the relationship between UTL and rate of warming was exponential. Solving this relationship for slow rates of warming used data across a range of warming scenarios and allowed the calculation of temperatures for long-term survival that equated to the acclimated state, and hence of value in predicting resistance to environmental warming.

UTLs at a warming rate of 1°C day^−1^ have recently been shown to vary between populations of species living in different parts of Antarctica, even though environmental temperature differences are small [23]. Both the clam *Laternula elliptica* and the starfish *Odontaster validus* had higher UTL values from the Antarctic Peninsula where summer temperatures range between 0°C and 1.5°C than in McMurdo Sound where temperatures rarely exceed 0°C. This mirrored data for fish species from the two locations [24] and showed that very small changes in annual temperature fluctuation in stenothermal species from environments of low temperature fluctuation produce clear effects in phenotypic plasticity.

Compared to adults, juveniles are considered generally less resilient to environmental stressors [3] including temperature [11], and reduced pH [25]. We address this paradigm by testing temperature tolerances in early juveniles and adults of four species of Antarctic marine invertebrates at different rates of thermal challenge. Antarctic marine ectotherms are a particularly good test system for understanding thermal sensitivity/resilience as they are stenothermal and have amongst the poorest abilities to resist warming [19], [26]-[[29], and this may be related to either the absence of, or reduced capacity in the heat shock response [30]. The most sensitive species identified to date, the brittle star *Ophionotus victoriae,* is incapable of surviving more than a few months at 2°C, a temperature less than 0.5°C above experienced summer maximum temperatures [31]. Survival margins, measured as the difference between temperatures tolerated in experimental systems compared to maximum or average experienced environmental temperatures are significantly lower than those for temperate species [21]. This is against a background of increasing climate temperatures, where the ocean to the West of the Antarctic Peninsula warming at one of the fastest rates on Earth, with sea temperatures having risen by 1°C in 50 years [32]. Hence there is a pressing need to understand species’ phenotypic plasticity in relation to elevated temperature in this region.

The aims of this study were to evaluate the UTLs of early juveniles compared to adults and to further evaluate effects of different rates of experimental warming. Comparisons were made for four different species and were tested at three different thermal regimes. The species chosen are all widespread, common, and locally abundant to very abundant and important components of the benthic ecosystem. *Odontaster validus* is a scavenger/predator seastar with a wide diet [33] and inhabiting a depth range from the intertidal to over 900 m, but is commonest from 15–200 m depth [34]. *Sterechinus neumayeri* is the most common Antarctic shallow water sea urchin. It also has a cosmopolitan diet that varies with substratum [35], and occurs over a wide depth range, although it is replaced by its sister species *S. antarcticus* at depths beyond around 400 m [36]. *L. elliptica* is the largest Antarctic bivalve mollusc. It is an infaunal suspension feeder and is a critical transformer of carbon from pelagic to benthic systems. The sea cucumber here, *Cucumaria georgiana* is also a suspension feeder, but lives attached to rocky substrata and macroalgae.

## Materials and Methods

### Animal Collections and Holding Conditions

All animals used in this study were collected under the relevant permits through the Foreign and Commonwealth Office of the United Kingdom. Specimens of all species studied were collected by hand by scuba divers from depths between 10 m and 20 m at sites near the British Antarctic Survey station at Rothera Point, Adelaide Island, Antarctic Peninsula (67°34′07″S, 68°07′30″W). Animals were collected carefully and kept underwater throughout their transfer from the sea to the station’s throughflow aquarium system. Following deposition in the aquarium animals were held in ambient temperature conditions for at least 7 days prior to use in experiments to ensure recovery from any stress effects from collection. Large specimens of *O. validus* and *S. neumayeri* were collected free ranging on various substrata. There has been recent work showing multiple species of the genus *Odontaster* live along the Antarctic Peninsula [37]. We therefore barcoded 305 specimens from the site studied here. All animals had the same COI sequence with occasional single base pair variation, at a level compatible with allelic variation. Large and small *L. elliptica* were dug by hand from sediment. Large and small *C. georgiana* were found in a red algal mat, largely comprised of *Phyllophora antarctica* that was common on broken rock substrata. Small juvenile *O. validus* and *S. neumayeri* were also present in the red algal mat and these were held with *P. antarctica* throughout, with the macroalga being regularly replenished during the longer term experiments. Experiments on *S. neumayeri* were conducted in the austral winter 2011 (August), and on *O. validus* in the summer 2012 (February). Experiments on the suspension feeding *L. elliptica* and *C. geogiana* were carried out in winter (August/September), when phytoplankton levels are amongst the lowest recorded on Earth, with chlorophyll levels below 0.01 mg m^−3^ [38]. Because of this no extra food was supplied beyond that entering the unfiltered aquarium water. For the scavengers *S. neumayeri* and *O. validus* food was offered as limpet tissue at approximately 5 day intervals. Winter temperatures on collection, and during experiments ranged between −1.5°C and −1.9°C and summer temperatures between 0.0°C and 0.9°C.

### Animal Size

In this study we used early juvenile or juvenile stages. For the seastar and urchins juveniles used were the previous season’s recruited cohort. The smallest juveniles used were identified and transferred to experimental systems using a low power binocular microscope with cold light in a controlled temperature room. During this process temperatures were monitored regularly and did not rise above 2°C at any time, and reached this value for only a few minutes. For the clam, ages ranged from less than 1 to between 1 and 2 years, based on shell growth rings. Ages for *C. georgiana* were harder to estimate, because they lack hard body parts, and knowledge of the size structure of the population is limited. The juveniles used were, however, very small, and adults were between 50 and 160 times heavier than the juveniles used (Table 1).

**Table 1:** Size data for adult and juvenile groups used in temperature tolerance evaluations for 4 species assessed in 1°C day^−1^ trials and 3 species in 1°C 3 days^−1^ trials.

	*L. elliptica*		*S. neumayeri*		*C. georgiana*		*O. validus*	
	Adult(lth, mm)	Juvenile(lth, mm)	Adult(D, mm)	Juvenile(D, mm)	Adult(wt, g)	Juvenile(wt, g)	Adult(wt, g)	Juvenile(wt, g)
1°C h^−1^								
Mean	75.16	15.33	36.33	2.87	8.02	0.049	12.63	0.107
s.e.	3.41	1.11	1.03	0.091	0.491	0.006	1.07	0.025
Max	100.5	23.3	47.7	3.8	15.3	0.138	22.41	0.55
Min	42.6	6.5	25.7	2.1	3.07	0.010	5.28	0.018
N	25	25	25	25	25	25	20	22
1°C day^−1^								
Mean	66.18	13.75	32.67	5.11	5.52	0.036	12.2	0.095
s.e.	1.87	0.80	0.73	0.51	0.437	0.003	1.57	0.013
Max	87.5	20.5	40.3	9.8	10.1	0.073	33.0	0.206
Min	46.4	8.2	25.6	1.4	1.80	0.012	4.35	0.017
N	25	24	25	25	25	28	20	19
1°C 3 days^−1^								
Mean	73.6	15.3	37.1	2.95	4.22	0.058		
s.e.	1.5	0.047	0.923	0.298	0.464	0.007		
Max	84.9	20.4	46.5	6.00	10.3	0.157		
Min	61.3	10.5	28.7	1.44	0.714	0.006		
N	24	26	25	24	25	25		

### Warming Experiments

Experiments were all conducted in the Bonner Laboratory at Rothera Station on Adelaide Island. Temperature control methods and regimes used were similar to those of [19], [39]. Specimens were placed in 75 L internal volume tanks with hollow walls, through which water was pumped from a temperature-controlled unit. A mixture of 25% alcohol in water was circulated through this system to provide fine scale temperature control. The whole system was placed inside a temperature-controlled room. Temperatures in this system could be held at a required set value ±0·1°C. After transfer to the experimental system animals were allowed 48 h to acclimatize to the new conditions. Thereafter temperatures were raised at the rate required for the relevant experimental protocol. For 1°C h^−1^ trials temperatures were raised at hourly intervals and animals checked at between 10 min and 20 min intervals. For 1°C day^−1^ trials temperatures were raised in 0.5°C steps on the morning and evening of each day and animals checked 3–5 times per day. This gave a gradual temperature rise across the day during experiments. For 1°C 3 days^−1^ trials temperatures were raised by ∼0.33°C day^−1^ and animals checked 3–5 times per day. In the latter two treatments water changes were routinely made twice weekly, but at 2 day intervals when temperatures were at the high end of the experimental range. Experiments always started at ambient temperature, and experiments at different rates of warming were carried out simultaneously in 3 separate systems of 1 tank per treatment and with adults and juveniles in the same tank. Temperature limit data were not collected at the slowest warming rate of 1°C 3 days^−1^ because of equipment failure.

Temperature limits were identified using either tactile stimuli (touching or prodding with a seeker), or appropriate behavioural stimuli (e.g. movement of tube feet or tentacles). For *L. elliptica* a lack of contraction in response to touching the sensory tentacles on the siphon was the criterion used; for *O. validus* the ambulacral groove was used; for *S. neumayeri* the lip around the mouth was used; and in *C. Georgiana* the ambulacral podia were stimulated. When animals were no longer responsive they were deemed to have reached their UTL. At this point for each individual the temperature was noted, and a measure of size made. For *L. elliptica* and *S. neumayeri* this was maximum shell or test dimension (measured with vernier callipers ±0.1 mm); for *C. georgiana* and *O. validus* this was surface dried (tissue blotted) wet weight (±0.001 g).

Initially all temperature limit data were tested for normality using Kolmogorov-Smirnov tests. Data for all species at all temperatures were not significantly different from normal distributions except for adult *C. georgiana* at 1°C h^−1^ (KS = 0.291, n = 24, P<0.01) and 1°C day^−1^ (KS = 0.048, n = 25, P = 0.048). These data were still significantly different from normal after logarithmic, double logarithmic and arcsin transformations. Where data were normally distributed (*L. elliptica*, *S. neumayeri* and *O. validus*), tests for homogeneity of variance were conducted using Levene’s test and no cases were significantly differences obtained (all values>2.99, P>0.09). Subsequently GLM analyses were carried out with post-hoc Tukey tests (Tukey-Kramer in unbalanced cases). For *C. georgiana* non-parametric Kruskal Wallis tests were used to identify overall factor effects (adult vs juvenile or warming rate) for the *C. georgiana* data and the H statistic quoted. Mann-Whitney tests were then used to compare sample medians and the W statistic quoted.

## Results

### Animal Sizes

In *S. neumayeri* large adults were on average 6 times larger in diameter than the small juveniles used, and in *L. elliptica* values for adults ranged from just over 6 to 12.6 times greater in length than those for juveniles (Table 1). In the soft bodied species *C. georgiana* adults were on average from around 50 times to 160 times heavier than the juveniles used, and in *O. validus* these values were 118 times to 126 times. The overall differences in size of adults and juveniles in the 4 species were thus similar. With isometric scaling a 6 fold increase in linear dimension equates to around a 200 times increase in weight. In all cases the specimens chosen were selected to represent mature adults and the earliest juvenile stages possible. Dissections confirmed the absence of gonads in juveniles and the presence of gonads in the adults of all species used. However, none of the adults appeared gravid, and in the three species where reproductive cycles have been studied, *L. elliptica, S. neumayeri* and *O. validus* experiments were not conducted during or prior to the spawning period [40]-[42].

### Temperature Limits

For *L. elliptica*, *S. neumayeri* and *O. validus* GLM analyses showed that there were significant adult vs juvenile, rate of warming and interaction effects on UTLs in all 3 species (Table 2).

**Table 2:** GLM statistics for effects of life history stage (adult vs juvenile), rate of warming and interaction terms on the upper temperature limits in *L. elliptica*, *S. neumayeri* and *O. validus*.

Species	Adult vs juvenile			Rate of warming			Interaction effect		
	F	d.f.	P	F	d.f.	P	F	d.f.	P
*L. elliptica*	8.09	1,142	0.005	578.9	2,142	<0.001	37.1	2,142	<0.001
*S. neumayeri*	35.9	1,142	<0.001	561.4	2,142	<0.001	6.25	2,142	0.002
*O. validus*	8.87	1,77	0.004	2294	1,77	<0.001	5.52	1,77	0.021

**Figure 1:**
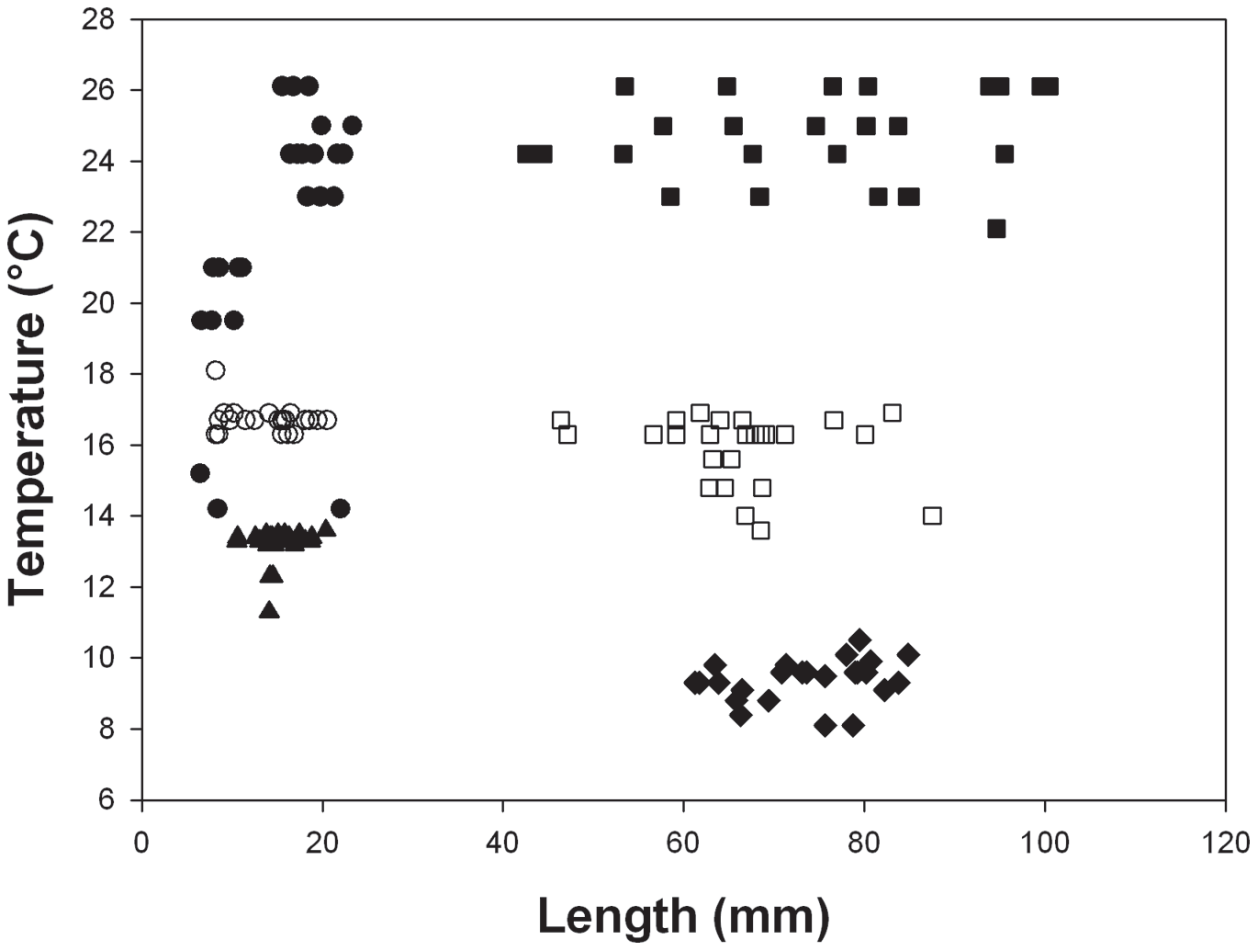
Upper temperature limits vs shell length for *L. elliptica*, at different warming rates. Data points shown are for individuals. At 1°C h^−1^• = juveniles, ▪ = adults; at 1°C day^−1^ ○ = juveniles, □ = adults; and at 1°C 3 days^−1^▴ = juveniles, • =  adults.

For *L. elliptica* Tukey post-hoc values were greater than 4.4 (P<0.001) for all comparisons, except adults vs juveniles at 1°C day^−1^, where juvenile and adult values were not significantly different (Tukey, T = 1.56, P = 0.63) (Fig. 1). Thus UTLs were lower at slower rates of warming in both adults and juveniles. Overall limits ranged from 14.2°C to 26.5°C at a warming rate of 1°C h^−1^, whereas they were 8.0°C to 13.6°C at 1°C 3 days^−1^. At the fastest rate of warming (1°C h^−1^) adults survived to higher temperatures than juveniles, at 1°C day^−1^ there was no difference and at 1°C 3 days^−1^ juveniles survived to higher temperatures than adults.

**Figure 2:**
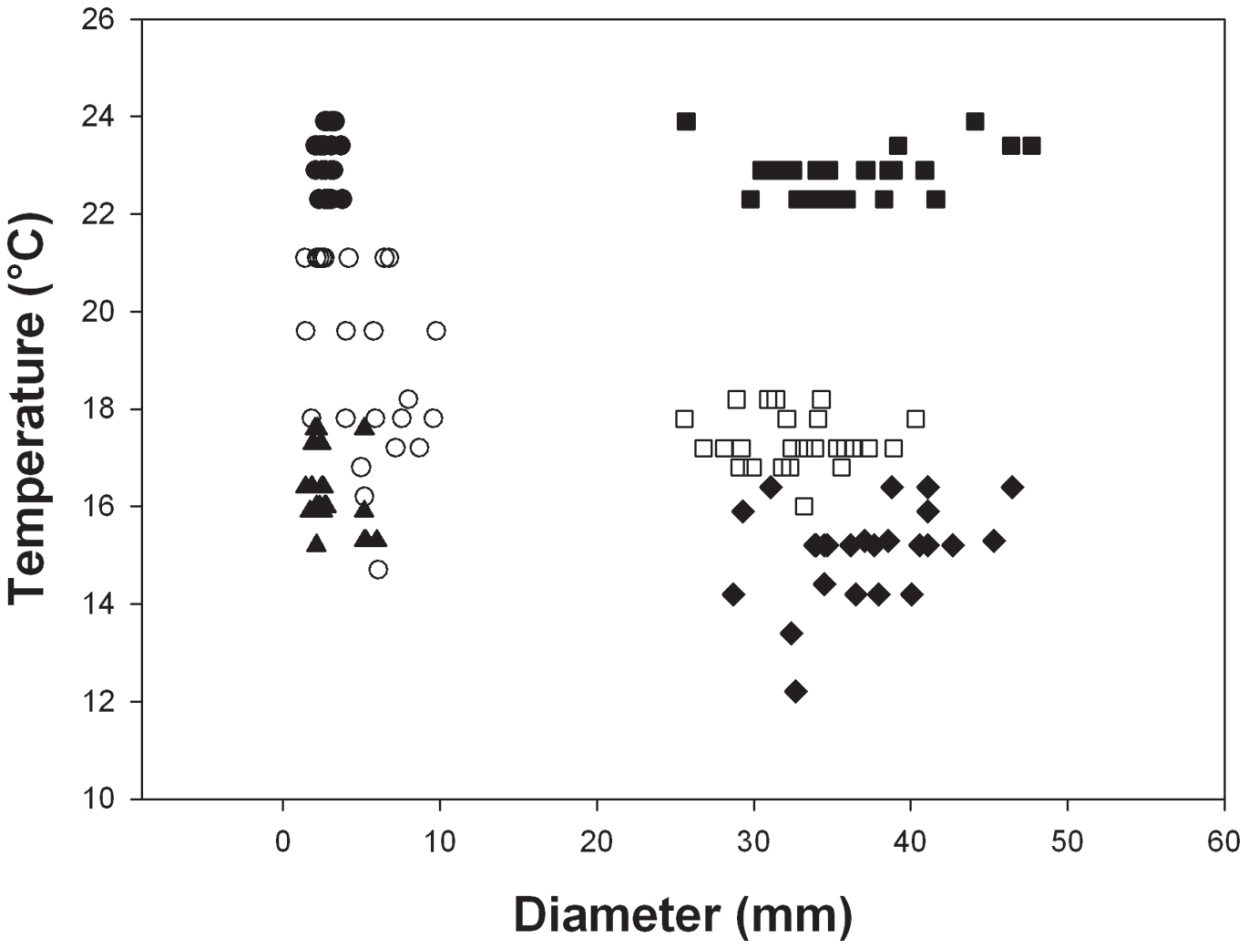
Upper temperature limits vs shell length for *S. neumayeri*, at different warming rates. At 1°C h^−1^• = juveniles, ▪ = adults; at 1°C day^−1^ ○ = juveniles, □ = adults; and at 1°C 3 days^−1^▴ = juveniles, • =  adults.

For *S. neumayeri* Tukey post hoc values were all greater than 3.2 (P = 0.02) except the comparison between adults and juveniles at the fastest rate of warming (1°C h^−1^) where there was no significant difference (T = 0.74, P = 0.98) (Fig. 2). As for *L. elliptica* UTLs were significantly lower at slower rates of warming. At 1°C h^−1^ values ranged from 22.2°C to 24.0°C, whereas at 1°C 3 days^−1^ these values ranged from 12.3°C to 17.6°C. Juveniles had higher UTLs than adults at the slowest 2 rates of warming (Fig. 2), but there was no difference between juveniles and adults at the fastest rate of 1°C h^−1^.

**Figure 3:**
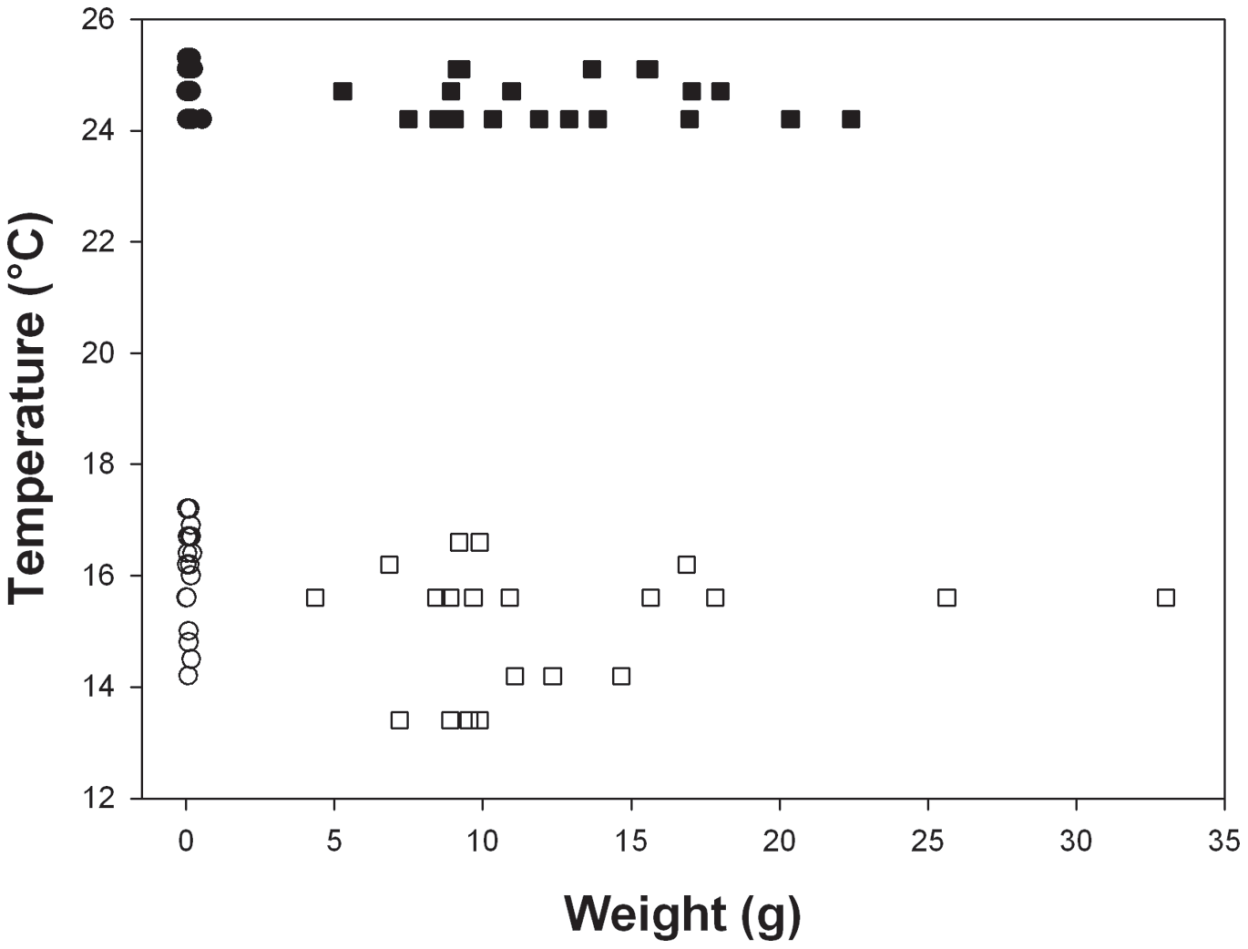
Upper temperature limits vs shell length for *O. validus*, at different warming rates. °C h^−1^• = juveniles, ▪ = adults; at 1°C day^−1^ ○ = juveniles, □ = adults; and at 1°C 3 days^−1^▴ = juveniles, • =  adults.

Tukey’s post-hoc comparisons for *O. validus* showed that juveniles survived to higher temperatures than adults at 1°C day^−1^(Fig. 3), but there was no difference at the faster rate of 1°C h^−1^ (T = 0.45, P = 0.97). Again temperature limits were lower at the slower rate of warming. Data were not collected at a warming rate of 1°C 3 days^−1^, but at 1°C h^−1^ UTLs ranged from 24.0°C to 25.3°C, and at 1°C day^−1^ the range was 13.7°C to 17.2°C.

**Figure 4:**
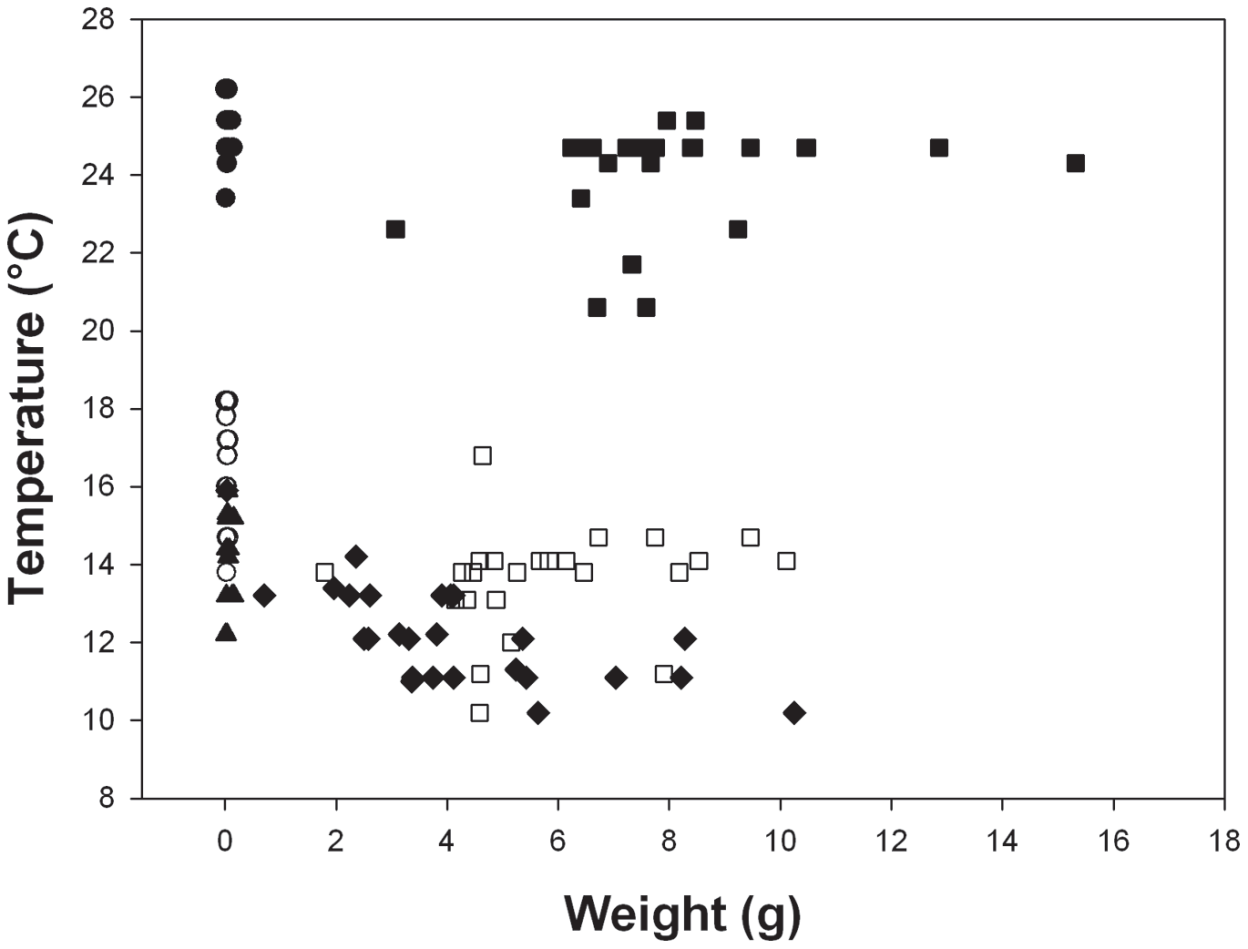
Upper temperature limits vs shell length for *C. georgiana*, at different warming rates. At 1°C h^−1^• = juveniles, ▪ = adults; at 1°C day^−1^ ○ = juveniles, □ = adults;and at 1°C 3 days^−1^▴ = juveniles, • =  adults.

Temperature limit data for *C. georgiana* varied significantly with life history stage (H = 24.0, 1 d.f., P<0.0001) and also with warming rate (H = 108.4, 2 d.f., P<0.0001). At all rates of warming juvenile *C. georgiana* had higher UTLs than adults (Fig. 4) (in all cases W>831, N≥49, P<0.0001). In both adults and juveniles UTLs at the fastest warming rate of 1°C h^−1^ were higher than at 1°C day^−1^, which were higher than those at 1°C 3 days^−1^ (W>325, N≥49 P<0.0001).

## Discussion

The results here do not fit with the paradigm of early stages being less resistant to stressors, as the juveniles studied survived to significantly higher temperatures than the adults in three out of four species at the warming rate of 1°C day^−1^ and in all species at 1°C 3 days^−1^ (Figs. 1–4, Table 2). At the fastest rate used (1°C h^−1^) juveniles survived to higher temperatures than adults in one species, adults to higher temperatures than juveniles in another and in the third and fourth species there was no difference. Hence, there was no overall difference between the two life history stages at a warming rate of 1°C h^−1^. The scale of the differences at the slower rates of warming was also surprising with early juveniles surviving to between 1°C and 5°C higher than adults (Figs. 1–4). Although juvenile survival to higher temperatures does not fit the general paradigm of greater sensitivity of earlier stages it has been suggested to fit with the oxygen and capacity limitation hypothesis [22], where a reduction in aerobic scope with age and hence a reduction in thermal window has been suggested to be a possible underlying principle [20], [43]. Running contrary to this, however, is that work on some groups, including fish has demonstrated an increase in aerobic scope with size and age from larvae to large adults [44]. On this basis an increased aerobic scope with age should increase thermal windows and not decrease them with age. Furthermore, smaller species have lower aerobic scopes than large species [45]. The reason for the reduction in thermal window in older animals thus remains obscure, but it may be that rising temperature reduces aerobic scope more rapidly in larger than in smaller animals. If the oxygen supply cascade scales isometrically, then larger animals may be less efficient at supplying oxygen to the mitochondria, needing a much longer path length for supply from the site of oxygen uptake [46], which could explain their increased sensitivity. Alternatively the maximum rate of oxygen uptake at respiratory surfaces usually scales as the square of linear dimension, whereas body mass scales at the cube [47], and metabolic rate predominantly scales with coefficients above the ⅔ that would represent isometric scaling of surface area to biomass. Thus, when animals are warmed, scaling of respiratory surfaces in relation to tissue volumes may cause tissues in larger animals to enter an anaerobic state earlier than those of animals in early life history stages. This situation may be further exacerbated by the observations in a number of species (including the clam, *L. elliptica* described here) that mitochondrial functions decline with age [48] and that older animals exhibit a larger stress response than juveniles when warmed [49]. Therefore utilisation of oxygen may be less efficient when it reaches the mitochondria in older, larger animals.

Antarctic marine species have significantly lower metabolic rates than related species from warmer latitudes [50]. Thus adaptation to low constant temperatures has, however, also resulted in species with low aerobic scopes [26], [51], which has been used to explain their extreme sensitivity to elevated temperature [20]. There is now a clear need to quantify aerobic scopes across life history stages and species where their relative temperature tolerances are known.

The data here showed that juveniles were more resistant to warming at rates of 1°C day^−1^ and 1°C 3 days^−1^, but not at 1°C h^−1^. This difference suggests that the mechanism setting UTLs at the more rapid warming used here is different from that at the slower rates. For Antarctic species reducing ambient oxygen levels has been shown to lower the critical temperatures in clams and small individuals maintain activity levels at higher temperatures than large ones when warmed [52]. The work supporting this paradigm is generally from experiments where the rate of warming has been between 1°C day^−1^ and 1°C every 2 weeks, but such experiments have not been carried out at the fastest rate of change of 1°C h^−1^ used here. The relationships between the UTL here for both juveniles and adults were lower at slower rates of warming. This is consistent with earlier studies where the reduction in UTL values with slowed rate of warming has been demonstrated to be exponential for 14 species of Antarctic marine ectotherms [19], for 34 species of tropical ectotherms from intertidal and subtidal habitats [22], and also for a range of marine invertebrate species from different latitudinal regions by [21]. The latter modelled the curves produced and calculated an asymptotic value for the long-term upper temperature limit (T_s_) that a species at a given site can survive to. By comparing T_s_ with the maximum experienced environmental temperature they calculated the spare capacity that exists between maximum experienced temperature and the long-term survival limit, which was termed the warming allowance (WA). This is low in Antarctic species, around 2–3°C on average across species (but <1°C in the most sensitive), but higher in temperate species at around 4–6°C [21]. Interestingly the WA for species in an El Nino affected area off Peru was large in years without an El Nino, but around zero during the event, when there were also large mortalities. [22] showed that the WA for tropical marine species is also low, around 2–3°C on average across many species.

The data presented here provide an intriguing counter to the argument that early life history stages are more sensitive to stresses and challenges when compared with adults and certainly promote the requirement for further investigations into the vulnerability (or resilience) of different life history stages. These data represent only four species, and expanding the species range will almost certainly reveal added complexities and emphasize that the temporal element is very important [53]. In barnacles, resistance to desiccation increases with age following settlement of cyprids [54], and older animals are more resilient than juveniles [55]. However, in molluscs age-related declines in muscle mass, mitochondrial efficiency, antioxidant activity, cell damage parameters and immune functioning have been demonstrated [49], [56], [57], which may contribute to reduced abilities to resist changing environmental conditions and potentially play a role in the reduced temperature tolerances in adults of at least one of the species described here. Further to this, studies showing longer survival for adults may suffer from interpretation problems in some cases. For instance a stress that impairs cell division, or cellular homeostasis, such as elevated temperature, lowered pH, or lowered salinity beyond critical values may result in adults surviving for extended periods while early stages fail more rapidly, because of the increased cell division rate in early stages. The stress would, however, be beyond the capacity for survival of both stages, and hence equally deleterious. The timescales of effects will differ across life history stages depending on a range of factors intrinsic to each stage. There is a clear need for more detailed comparisons of resilience to stresses across the whole range of life history stages.

The results here, in addition to those already discussed, emphasize that at every stage in the life history of a species, there are constraints and trade-offs [58], [59], the impacts of which may differ according to the age of the animal, irrespective of any proposed overarching limitations, such as oxygen supply. Numbers present in each category are limited, but the general patterns of decreasing UTL at slower rates of warming and juveniles surviving to higher temperatures than adults at rates of warming of 1°C day^−1^ and slower, were consistent across species and hence trophic guilds. This agreed with the finding of [19] who demonstrated that there was no relationship between UTL and trophic guild for 14 species of benthic marine invertebrates when warmed at 1°C day^−1^. These data for inter-species comparisons are limited and must be interpreted with caution, but differential survival of species with different activity levels would have powerful consequences for the structure of the benthic ecosystem.

## References

[1] Vernon HM (1900) The death temperature of certain marine organisms. J Physiol 25: 131–136. 10.1113/jphysiol.1899.sp000782 PMC1516668 16992521

[2] Stokes MD, Holland ND (1996) Life history characteristics of the Florida lancelet, *Branchiostoma floridae*: some factors affecting population dynamics in Tampa Bay. Israel J Zool 42 (Suppl.): 67–86.

[3] Spicer JI, Gaston KJ (1999) Physiological Diversity and its Ecological Implications. Oxford: Blackwell Science. 241 pp.

[4] Gosselin LA, Qian PY (1997) Juvenile mortality in benthic marine invertebrates. Mar Ecol Prog Ser 146: 265–282.

[5] Pechenik JA (1999) On the advantages and disadvantages of larval stages in benthic marine invertebrate life cycles. Mar Ecol Prog Ser 177: 269–297.

[6] Atkinson D (1996) Ectotherm life-history responses to developmental temperature. !n: Johnston IA, Bennet AF, editors. Animals and Temperature: phenotypic and evolutionary adaptation. Society for Experimental Biology Seminar Series, 59. Cambridge: Cambridge University Press. 183–204.

[7] Pearse JS, McClintock JB, Bosch I (1991) Reproduction of Antarctic benthic marine-invertebrates - tempos, modes, and timing. Am Zool 31: 65–80.

[8] Stanwell-Smith DP, Peck LS (1998) Temperature and embryonic development in relation to spawning and field occurrence of larvae of 3 Antarctic echinoderms. Biol Bull Woods Hole 194: 44–52. 10.2307/1542512 28574786

[9] Huntsman AG (1925) Limiting factors for marine animals. 2. Resistance of larval lobsters to extreme temperatures. Contr Can Biol 2: 91–93.

[10] Sulkin SD, Mojica E, McKeen GL (1996) Elevated summer temperature effects on megalopal and early juvenile development in the Dungeness crab, *Cancer magister* . Can J Fish Aquat Sci 53: 2076–2079.

[11] Gosselin LA, Chia FS (1995) Characterising temperate rocky shores from the perspective of an early juvenile snail: the main threats to survival of newly hatched *Nucella emarginata* . Mar Biol 122: 625–635.

[12] Pörtner HO, Knust R (2007) Climate change affects marine fishes through the oxygen limitation of thermal tolerance. Science 315: 95–97. 17204649 10.1126/science.1135471

[13] Chapelle G, Peck LS (1999) Polar gigantism dictated by oxygen availability. Nature 399: 114–115.

[14] Chapelle G, Peck L (2004) Amphipod crustacean size spectra: new insights in the relationship between size and oxygen. Oikos 106: 167–175.

[15] Peck LS, Chapelle G (2003) Reduced oxygen at high altitude limits maximum size. Proc R Soc Lond BL 270: S166–S167. 10.1098/rsbl.2003.0054 PMC1809933 14667371

[16] Angilletta MJ Jr, Dunham AE (2003) The temperature-size rule in ectotherms: simple evolutionary explanations may not be general. Am Nat 162(3): 332–42. 12970841 10.1086/377187

[17] Pankhurst NW, Munday PL (2011) Effects of climate change on fish reproduction and early life history stages. Mar Freshwat Res 62: 1015–1026.

[18] Lo-Yat A, Meekan MG, Lecchini D, Martinez E, Galzin R (2011) Extreme climatic events reduce ocean productivity and larval supply in a tropical reef ecosystem. Glob Change Biol 17: 1695–1702.

[19] Peck LS, Clark MS, Morley SA, Massey A, Rossetti H (2009) Animal temperature limits and ecological relevance: effects of size, activity and rates of change. Funct Ecol 23: 248–253.

[20] Pörtner HO, Peck LS, Somero GA (2007) Thermal limits and adaptation in marine Antarctic ectotherms: an integrative view. Philos Trans R Soc Lond B 362: 2233–2258. 17553776 10.1098/rstb.2006.1947 PMC2443174

[21] Richard J, Morley SA, Thorne MAS, Peck LS (2012) Estimating long-term survival temperatures at the assemblage level in the marine environment: towards macrophysiology. PLoS One 7: e34655. 22509340 10.1371/journal.pone.0034655 PMC3324497

[22] Nguyen KDT, Morley SA, Lai C-H, Clark MS, Tan KS, et al (2011) Upper Temperature Limits of Tropical Marine Ectotherms: Global Warming Implications. PLoS One 6(12): e29340. 22242115 10.1371/journal.pone.0029340 PMC3248430

[23] Morley SA, Martin SM, Bates AE, Clark MS, Ericson J, et al (2012) Spatial and temporal variation in the heat tolerance limits of two abundant Southern Ocean invertebrates. Mar Ecol Prog Ser 450: 81–92.

[24] Bilyk KT, DeVries AL (2011) Heat tolerance and its plasticity in Antarctic fishes. Comp Biochem Physiol A 158: 382–390. 10.1016/j.cbpa.2010.12.010 21159323

[25] Byrne M (2011) Impact of ocean warming and ocean acidification on marine invertebrate life stages: vulnerabilities and potential for persistence in a changing ocean. Oceanogr Mar Biol Annu Rev 49: 1–42.

[26] Peck LS, Conway LZ (2000) The myth of metabolic cold adaptation: oxygen consumption in stenothermal Antarctic bivalve molluscs. In: Harper E, Crame AJ, editors. Evolutionary biology of the bivalvia. Geological Society of London Special publication 177. Cambridge: Cambridge University Press. 441–450.

[27] Peck LS, Morley SA, Clark MS (2010) Poor acclimation capacities in Antarctic marine ectotherms. Mar Biol 157: 2051–2059.

[28] Pörtner HO (2002) Physiological basis of temperature-dependent biogeography: trade-offs in muscle design and performance in polar ectotherms. J Exp Biol 205: 2217–2230. 12110656 10.1242/jeb.205.15.2217

[29] Somero GN, DeVries AL (1967) Temperature tolerance of some Antarctic fishes. Science 56: 257–258. 10.1126/science.156.3772.257 6021046

[30] Clark MS, Peck LS (2009) HSP70 Heat shock proteins and environmental stress in Antarctic marine organisms: a mini-review. Mar Gen 2: 11–18. 10.1016/j.margen.2009.03.003 21798167

[31] Peck LS, Massey A, Thorne M, Clark MS (2009) Lack of acclimation in *Ophionotus victoriae*: brittle stars are not fish. Pol Biol 32 399–402.

[32] Meredith MP, King JC (2005) Rapid climate change in the ocean west of the Antarctic Peninsula during the second half of the 20^th^ century. Geophys Res Letters 32: L19604–L19609.

[33] Pearse JS (1966) Antarctic asteroid *Odontaster validus*: Constancy of reproductive periodicities. Science 15: 1736–1764. 10.1126/science.152.3730.1763 17757799

[34] Dearborn JH (1977) Foods and Feeding Characteristics of Antarctic Asteroids and Ophiuroids. In: Llano GA, editor. Adaptations within Antarctic ecosystems: Proceedings of the third SCAR symposium on Antarctic biology. Houston: Gulf Publishing. 293–326.

[35] McClintock JB (1994) Trophic biology of Antarctic shallow water echinoderms. Mar Ecol Prog Ser 111: 191–202.

[36] Brey T, Gutt J (1991) The genus *Sterechinus* (Echinodermata: Echinoidea) on the Weddell Sea shelf and slope. Pol Biol 11: 227–232.

[37] Janosik AM, Mahon AR, Halanych KM (2011) Evolutionary history of Southern Ocean Odontaster sea star species (Odontasteridae; Asteroidea). Pol Biol 34: 575–586.

[38] Clarke A, Meredith MP, Wallace MI, Brandon MA, Thomas DN (2008) Seasonal and interannual variability in temperature, chlorophyll and macronutrients in northern marguerite bay, Antarctica. Deep Sea Res Part II 55: 1988–2006.

[39] Peck LS, Webb KE, Bailey D (2004) Extreme sensitivity of biological function to temperature in Antarctic marine species. Funct Ecol 18: 625–630.

[40] Brockington S, Peck LS, Tyler PA (2007) Gametogenesis and gonad mass cycles in the common circumpolar Antarctic echinoid *Sterechinus neumayeri.* . Mar Ecol Prog Ser 330: 139–147.

[41] Grange L, Peck LS, Tyler PA (2007) Multi-year observations on the gametogenic ecology of the Antarctic seastar *Odontaster validus.* . Mar Biol 153: 15–23.

[42] Peck LS, Powell DK, Tyler PA (2007) Very slow development in two Antarctic bivalve molluscs, the infaunal clam, *Laternula elliptica* and the scallop *Adamussium colbecki* . Mar Biol 150: 1191–1197.

[43] Pörtner HO (2004) Climate variability and the energetic pathways of evolution: the origin of endothermy in mammals and birds. Physiol Biochem Zool 77: 59–981. 10.1086/423742 15674770

[44] Killen SS, Costa I, Brown JA, Gamperl AK (2007) Little left in the tank: metabolic scaling in marine teleosts and its implications for aerobic scope. Proc R Soc Lond B 274: 431–438. 10.1098/rspb.2006.3741 PMC1702384 17164208

[45] Weibel ER, Bacigalupe LD, Schmitt B, Hoppeler H (2004) Allometric scaling of maximal metabolic rate in mammals: muscle aerobic capacity as determinant factor. Resp Physiol Neurobiol 140: 115–132. 10.1016/j.resp.2004.01.006 15134660

[46] Atkinson D, Morley SA, Hughes RN (2006) From cells to colonies: at what levels of body organisation does the “temperature size rule” apply? Evol Dev 8: 202–214. 16509898 10.1111/j.1525-142X.2006.00090.x

[47] Nielsen K (1997) Animal Physiology. 5^th^ edition. Cambridge: Cambridge University Press. 612 p.

[48] Philipp E, Pörtner HO, Abele D (2005) Mitochondrial ageing of a polar and a temperate mud clam. Mech Ageing Dev 126: 610–619. 15811430 10.1016/j.mad.2005.02.002

[49] Clark MS, Husmann G, Thorne MAS, Burns G, Truebano M, et al.. (2013) Response to environmental change: age as a significant factor in sensitivity to hypoxic stress. Glob Change Biol. doi:10.1111/gcb.12197.

[50] Clarke A, Johnston N (1999) Scaling of metabolic rate and temperature in teleost fish. J Anim Ecol 68: 893–905.

[51] Peck LS (2002) Ecophysiology of Antarctic marine ectotherms: limits to life. Pol Biol 25: 31–40.

[52] Peck LS, Morley SA, Pörtner HO, Clark MS (2007) Small increases in temperature and reductions in oxygen availability limit burrowing capacity in the Antarctic clam *Laternula elliptica* . Oecologia 154: 479–484. 17899201 10.1007/s00442-007-0858-0

[53] Peck LS (2011) Organisms and responses to environmental change. Mar Gen 4: 237–243. 10.1016/j.margen.2011.07.001 22118635

[54] Barnes RSK, Hughes RN (1988) An Introduction to Marine Ecology. 2^nd^ edition. Oxford: Blackwell Scientific Publications. 286 p.

[55] Foster BA (1971) Desiccation as a factor in the intertidal zonation of barnacles. Mar Biol 8: 12–29.

[56] Abele D, Brey T, Philipp E (2009) Bivalve models of aging and the determination of molluscan lifespans. Exp Geront 44: 307–315. 10.1016/j.exger.2009.02.012 19268513

[57] Husmann G, Philipp EER, Rosenstiel P, Vazquez S, Abele D (2011) Immune response of the Antarctic bivalve *Laternula elliptica* to physical stress and microbial exposure. J Exp Mar Biol Ecol 398: 83–90.

[58] Zera AJ, Harshman LG (2001) The physiology of life history trade-offs in animals. Annu Rev Ecol Syst 32: 95–126.

[59] Bayne BL (2004) Phenotypic flexibility and physiological tradeoffs in the feeding and growth of marine bivalve molluscs. Integr Comp Biol 44: 425–432. 21676728 10.1093/icb/44.6.425

